# Piezoelectric Energy Harvesting from Low-Frequency Vibrations Based on Magnetic Plucking and Indirect Impacts

**DOI:** 10.3390/s22155911

**Published:** 2022-08-08

**Authors:** Michele Rosso, Alessandro Nastro, Marco Baù, Marco Ferrari, Vittorio Ferrari, Alberto Corigliano, Raffaele Ardito

**Affiliations:** 1 Department of Civil and Environmental Engineering, Politecnico di Milano, 20133 Milano, Italy; 2 Department of Information Engineering, Università degli Studi di Brescia, 25123 Brescia, Italy

**Keywords:** combined actuation, magnetic plucking, indirect impacts, piezoelectric energy harvesting, nonlinear dynamics

## Abstract

This work proposes a mono-axial piezoelectric energy harvester based on the innovative combination of magnetic plucking and indirect impacts, e.g., impacts happening on the package of the harvester. The harvester exploits a permanent magnet placed on a non-magnetic mass, free to move within a predefined bounded region located in front of a piezoelectric bimorph cantilever equipped with a magnet as the tip mass. When the harvester is subjected to a low-frequency external acceleration, the moving mass induces an abrupt deflection and release of the cantilever by means of magnetic coupling, followed by impacts of the same mass against the harvester package. The combined effect of magnetic plucking and indirect impacts induces a frequency up-conversion. A prototype has been designed, fabricated, fastened to the wrist of a person by means of a wristband, and experimentally tested for different motion levels. By setting the magnets in a repulsive configuration, after 50 s of consecutive impacts induced by shaking, an energy of 253.41 μJ has been stored: this value is seven times higher compared to the case of harvester subjected to indirect impacts only, i.e., without magnetic coupling. This confirms that the combination of magnetic plucking and indirect impacts triggers the effective scavenging of electrical energy even from low-frequency non-periodical mechanical movements, such as human motion, while preserving the reliability of piezoelectric components.

## 1. Introduction

The growing need for the Internet of Things (IoT) has pushed research on microelectromechanical systems (MEMS) in the development of autonomous low-power sensors and actuators, intending to reduce battery consumption [1]. The great fertility of this sector is evidenced by the fact that the MEMS market was valued at USD 48.74 billion in 2018. Before the COVID-19 Pandemic, it was expected to grow up to USD 122.83 billion in 2026 with a compound annual growth rate (CAGR) of 11.3% [2].

A possible strategy for the creation of autonomous smart devices consists of exploiting the environmental kinetic energy and converting it into electrical energy through mechanical vibrations of a piezoelectric transducer [3,4,5]. The environmental kinetic energy is distributed over a low-frequency band (e.g., 0–100 Hz) [6,7] while piezoelectric transducers typically exhibit an effective dynamic amplification at hundreds or even thousands of Hertz. This is a very important drawback, especially in the field of human motion, that is ultra-low frequency and, in some cases, non-periodic [8]. To overcome this limitation, testified by many works in the scientific subset of linear piezoelectric resonant harvesters [6,9,10], nowadays the trend is to exploit physical or geometrical non-linearities for inducing frequency up-conversion. The most classic ways are referred to as multi-stability or impacts. Several investigations have been carried out on direct impacts, also, in recent times [11]. This approach has the advantage of easily handling low-frequency input and providing multi-modal structural responses. However, it suffers from the fact that it does not guarantee high reliability since piezoelectric materials are brittle. As an alternative, indirect impact has been studied in recent times. Halim and Park [12] presented a piezoelectric energy harvester up-converted via indirect impacts on flexible structures supporting unimorph transducers. A similar concept has been realized by Ju and Ji [13] with focus also on the long-term reliability. In the field of multi-stability, many researchers used snap-through buckling mechanisms of structures: Speciale et al. [14] designed a prototype of bistable arch that activates multi-modal responses of cantilever clamped to the keystone section; Xu et al. [15] proposed a prototype of MEMS harvester that operates below 100 Hz owing to a snap-through mechanism. Recently, some researchers solved the frequency conversion problem by widening the operating band of piezoelectric harvesters through sliding masses that tune the structural frequency at the input frequency. Shin et al. implemented this idea on a piezoelectric double-clamped harvester [16], and Wang et al. on a flute-inspired cantilever beam [17]. The drawback of these systems is that they need a continuous signal to reach steady state regime and they do not work in case of random movement. Some groups enhanced the electronics and developed advanced electronic techniques to increase the operational bandwidth or the scavenged energy. Bonnin et al. [18] designed an impedance matching network that can amplify the dynamics response of the harvester at a frequency chosen by the designer. Yan et al. [19], and Yu and Zhou [20] used the combination inductor-resistor to widen the frequency band. 

Another approach, widely studied in the literature, consists in exploiting the magnetic interaction for inducing a plucking phenomenon on the piezoelectric beams. The main advantage of this strategy is the contactless energy transfer without mechanical damage to the transducer. Magnetic plucking can be arranged both in the repulsive configuration of the magnets (multi-stable system) and in the attractive configuration (piecewise stiffening system). Many works have been presented in the framework of rotational mechanisms: Pillatsch et al. [21,22] realized studies and a prototype for applications on watches; numerical FE-based analyses of the magnetic plucking with experimental studies with rotors have been conducted by Dauksevicius et al. [23]; proof-of-concept of rotational devices in the field of wearable applications with imposed motion have been presented by Kuang et al. [24] in 2015 and by Pozzi et al. [25] in 2016 for knee-joint harvesters. With reference to translational mechanisms, recent investigations on translational actuating mechanisms have been proposed by Li et al. [26], in which a magnet is connected to spring for creating a bandwidth bistable device with amplitude variable potential wells, and by Baù et al. [27], who carried out numerical and experimental investigations on multi-frequency array of transducer interacting with a permanent magnet held or elastically fixed to the supporting base. 

The literature on frequency up-conversion via indirect impact is sharply separated with respect to the literature about magnetic plucking. The present paper introduces an innovative combination of the two mechanisms with the purpose of enhancing the performances of energy harvesting via piezoelectric transduction from low-frequency vibration is considered. More specifically, a meso-scale, wearable prototype is proposed with a piezoelectric bimorph cantilever equipped with a magnet as the tip mass. A slot hosts a mass that is freely movable in a single direction. The mass is non-magnetic but equipped with a permanent magnet. When the device is subjected to an external acceleration, the moving mass magnetically plucks the transducer and impacts the package with further vibrational input to the cantilever. This concept combines different techniques with promising reliable applications for human-limb motion.

The paper is organized as follows: in Section 2 the theory of operation is presented with a mathematical model. In Section 3 the prototype is presented and in Section 4 experimental investigations are summarized. Closing remarks are put forward in Section 5.

## 2. Description of the Piezoelectric Energy Harvester

**Figure 1:**
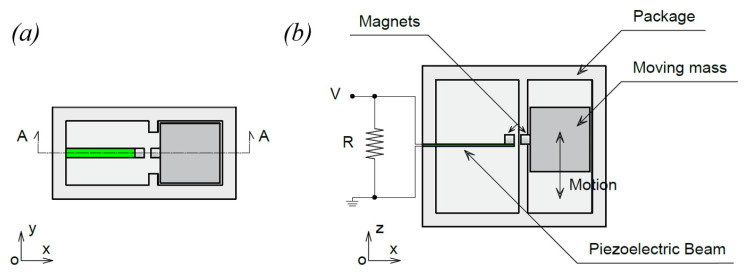
Conceptual illustration of the device: (**a**) xy plane view (**b**) section A-A view, plane xz.

**Figure 2:**
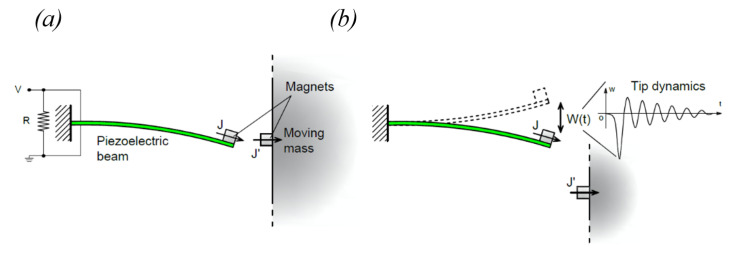
Illustration of the magnetic plucking mechanism: (**a**) the mass approaches the cantilever, (**b**) high frequency vibrations of the piezoelectric cantilever after the snap in case of attractive interaction.

The working principle of the proposed piezoelectric energy harvester is schematically represented in Figure 1a,b. It is composed of a piezoelectric beam and a non-magnetic moving mass constrained in a translational guide, both equipped with a permanent magnet. A permanent magnet is placed on the tip of the piezoelectric beam to achieve a double function: reduce the eigenfrequency of the beam and enable the magnetic interaction. Another permanent magnet is located on the abovementioned moving mass, disposed in front of the piezoelectric cantilever as indicated in Figure 1 and Figure 2. The magnets can be arranged both in attractive and repulsive configurations. In this work, parallel magnetization vectors *J* and *J*′ are considered, aligned with the longitudinal axis of the piezoelectric beam, as indicated in Figure 2a. With the represented orientation of *J* and *J*′ the magnetic force between the magnets is attractive. When the mass moves due to an external forcing input, the magnetic interaction between the two permanent magnets deforms the beam and, when the elastic force exceeds the magnetic one, a release of the beam happens with consequent free oscillations of the beam, as represented in Figure 2b by means of the tip displacement *W*(*t*). This phenomenon is called magnetic plucking [16]. In addition, when the mass collides with the harvester package the generated impact activates additional mechanical vibrations in the piezoelectric cantilever through the clamp. Therefore, the presence of the mass and the magnetic field interaction triggers the vibrations at the natural frequencies of the piezoelectric cantilever.

With the proposed combined mechanism, it is possible to harvest electrical energy from low-frequency non-periodical mechanical movements, while safeguarding the reliability of piezoelectric components since they are made of brittle materials. Furthermore, the hybrid mechanism guarantees that if the plucking does not occur, the harvester can operate with impacts only.

To understand the working principle of the proposed device, a simple mathematical model can be adopted. The piezoelectric beam and the mass can be modelled separately and then put in a global model coupled by the magnetic interaction and the impulsive effect of impacts. The equations of motion are derived from the classical Euler–Lagrangian approach in dissipative form. Both the kinematic field and the voltage of the piezoelectric cantilever are discretized via Rayleigh–Ritz method [28,29]. The displacement field is governed by a single time-dependent degree of freedom W(t), representing the tip beam displacement [30]. A single degree-of-freedom is also assumed for the electric field. Since the piezoelectric layers are very thin (280 μm) and extended along the whole length of the beam, a linear voltage is assumed along the thickness *t_p_* of the layer; moreover, in view of the presence of a unique electrode along the beam’s axis, the voltage does not depend on the x coordinate and is constant along that axis. The voltage V(t) at the electrode is the time-dependent degree of freedom. By applying the Euler–Lagrangian approach it is possible to obtain the governing differential system of ordinary differential equations (ODEs). Furthermore, the dynamics of the moving mass is described by the Newton’s second law applied to a rigid body subjected to an external acceleration, a magnetic force, and to the gravity field if the mechanism is placed to work in the vertical direction.

By considering the presence of the mass, the global dynamics is represented in the following differential system.

(1) $$\{\begin{array}{c} m\;\ddot{W}+c_{m}\;\dot{W}+k_{l}\;W-\theta \;V=-m_{z}\;\ddot{Z}+f_{mag}+f_{g}+f_{imp}\\ C_{e}\dot{V}+\theta \;\dot{W}+R^{-1}\;V=0\\ Ma=-M\;\ddot{Z}-f_{mag}+f_{G}-f_{imp} \end{array}$$

where *m* is the inertial coefficient, *c_m_* is the linear damping coefficient, $k_{l}$ is the linear elastic stiffness, *m_z_* is the activated mass [29] and $C_{e}$ is the capacitance equivalent to piezoelectric layers. θ is the coefficient related to the multiphysics coupling due to the piezoelectricity. The external force is, in this case, the sum of inertial forces due to vibrational input, the magnetic interaction force, the impact force and the gravity load in case the device works in the vertical direction. $\ddot{Z}$ is the input acceleration on the device, $f_{g}$ and $f_{G}$ are the gravity loads of the discretized cantilever and moving mass, respectively. $f_{mag}$ is the plucking magnetic force and *a* is the resulting acceleration due to Newton’s law on the moving mass and *M* is the moving mass. In system (1), the time rate of electrical charge is obtained under the hypotheses of the connection with a resistive load *R*, as common in energy harvesting investigations [1,4].

**Figure 3:**
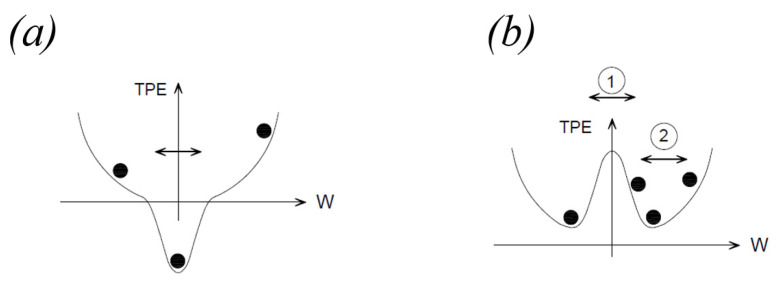
Qualitative total potential energy (TPE) function with respect to tip displacement W, for two versions of the system in Equation (1): (**a**) attractive configuration; (**b**) repulsive configuration with inter-well (1) and intra-well (2) mechanisms.

The analytical formulation of the plucking magnetic force has been largely disserted in the past years [31,32,33,34,35]. For the presence of the magnetic force, the system of Equation (1) becomes non-linear. For the attractive configuration of magnets, the system is mono-stable but the behavior changes when the distance *W* between the magnets exceeds a certain threshold, as reported in Figure 3a where the total potential energy (TPE) has been plotted as a function of *W*. Conversely, for repulsive configuration the system is bi-stable and two symmetric potential wells are present, see Figure 3b. As a consequence, intra-well (small) or inter-well (large) oscillations are possible. 

The system is non-linear also for the presence of indirect impacts $f_{imp}$: the impulses on a flexible structure can be analytically considered via power series for the forcing function as in [13] or by considering vibrations at the clamp as made in [12].

## 3. Design and Fabrication

**Figure 4:**
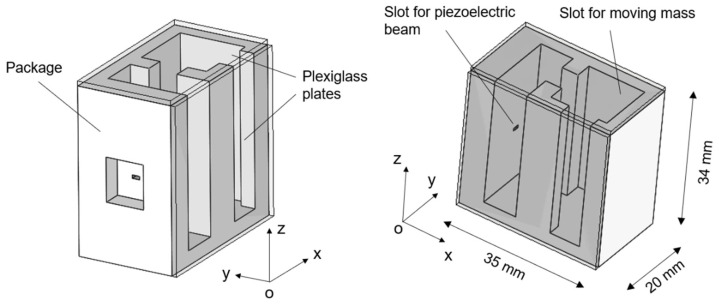
Illustration of the package of the piezoelectric energy harvester designed with Solidworks©.

**Figure 5:**
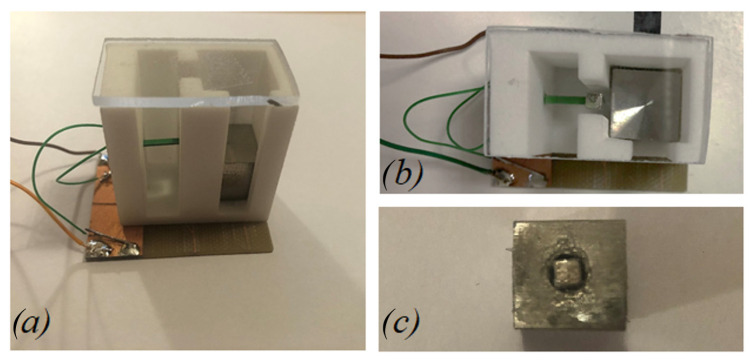
Assembled prototype (**a**) lateral and (**b**) top view. (**c**) Detail of the arrangement of the permanent magnet inside the moving mass.

The 3D structure of the mono-axial piezoelectric harvester is illustrated in Figure 4. The device is composed of a polyamide package, with a size of 35 × 34 × 20 mm^3^ in which there are two main slots used to host, respectively, the piezoelectric cantilever beam and the moving mass. Between the two slots, a free zone is used to set a gap distance between the magnets of less than 1.0 mm. The top and lateral faces of the device have been covered with a plexiglass plate, as shown in Figure 5a,b. The moving mass is a cube of 13 mm side length and with a mass of 0.017 kg. It is made of non-magnetic steel AISI 316 with the aim of not affecting the magnetic field provided by the equipped magnet. The Neodymium magnets are cubic with a 3 mm side length and with a magnetization of 1.32 T. The piezoelectric cantilever beam is a commercial bimorph element RS 285-784, RS Components^®^ with the active layers connected in series. Its features are summarized in Table 1: the titanium shim is enclosed in two PZT layers. Considering the clamped zone of the piezoelectric beam, the effective length is 10.5 mm. A copper foil on FR4 has been glued on the bottom face of the device orthogonal to the guide direction to weld the electrical connections.

## 4. Experimental Results

**Figure 6:**
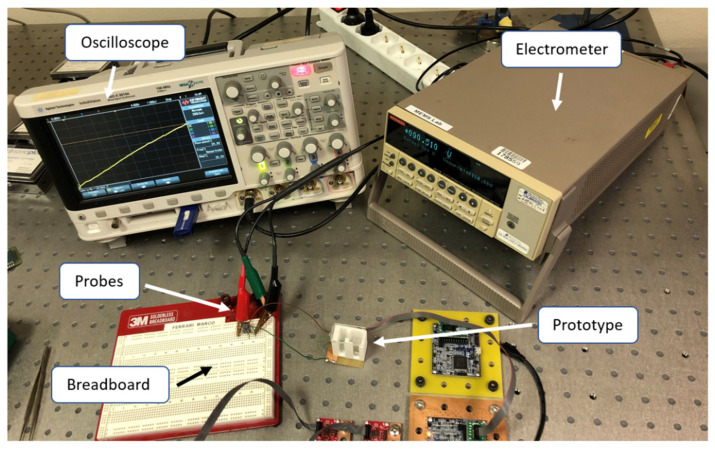
Experimental set up with indication of all the equipment and the prototype.

**Figure 7:**
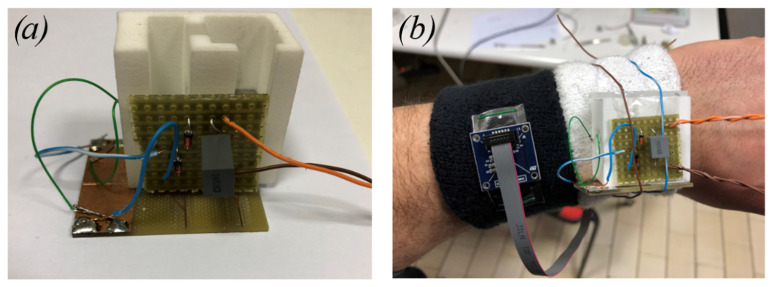
(**a**) Close-up of the rectifier circuit board. (**b**) Accelerometer and prototype tied to the wrist.

Experimental tests have been carried out to investigate the magnetic plucking mechanism, the effects of indirect impacts in realistic situations of human motion and the performance of the device under different magnetic configurations and electrical circuitry. Specifically, the combined effect of magnetic plucking and indirect impacts has been tested with the proposed device and compared to the case of indirect impacts only. In case of the presence of magnetic plucking, both the repulsive and the attractive configurations of the magnets have been considered. Figure 6 shows the experimental setup with the indication of the involved components, while Figure 7a shows the energy harvester connected to a passive diode-based voltage-doubler rectifier feeding a storage capacitor. Figure 7b depicts the harvester and a triaxial accelerometer (STMicroelectronics IIS3DWB) tied to the wrist of a person by means of wristbands. The accelerometer has been employed to monitor the accelerations to which the harvester is subjected. Experimental modal analysis has been performed for each condition of interaction between the moving mass and the piezoelectric beam.

### 4.1. Free Vibration of the Piezoelectric Beam

**Figure 8:**
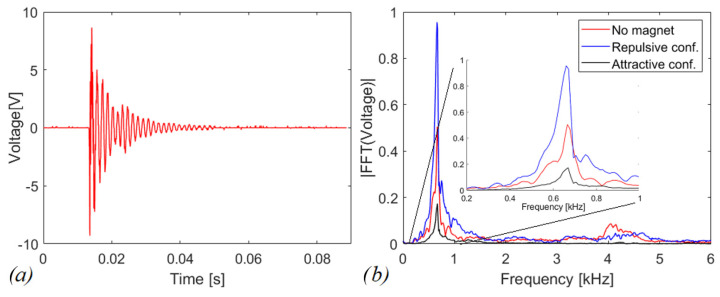
(**a**) Measured voltage across R = 100 kΩ due to free oscillations in case of a single indirect impact in absence of moving mass in the slot. (**b**) FFT responses of the open circuit voltage with a close-up on [0.2,1] kHz.

The dynamic behavior of the piezoelectric beam has been studied by measuring the free oscillations of the cantilever, with the magnet on its tip. The analysis is carried out in the electrical domain by measuring the voltage response to impulsive excitation and connecting the cantilever to a resistive test load of 100 kΩ. Data have been acquired by employing an oscilloscope Agilent MSOX3014A with internal input resistance R_0_ equal to 1 MΩ and a capacitance C_0_ of 14 pF. Figure 8a shows the voltage across the resistor as a function of time in case of a single indirect impact in the absence of moving mass in the slot. In this case, the indirect impact is generated by an external mechanical impulse imparted to the package. In other tests, the impulse is induced by the magnetic plucking, in attractive and repulsive configurations: the mass is pushed and forced to move in the slot, whose bottom is removed. Figure 8b reports the FFTs for all the aforementioned cases. The first eigenfrequency of the cantilever is 659.2 Hz, 665.2 Hz, and 668.3 Hz for repulsive, no magnetic interaction and attractive cases, respectively. Through a MATLAB^®^ program, in which the modelling of the piezoelectric cantilever has been implemented [35], an eigenfrequency of 672.5 Hz is obtained without interactions. This value is 1% larger than the experimental one, because of the discretization approach.

From the experimental data, the quality factor has been computed via the logarithmic decrement method [36] to have an idea how much the system is damped. By processing the 14 peaks of the voltage time histories in free oscillations, a value of Q_M_ = 13.72 is obtained without the magnetic interaction. 

### 4.2. Experimental Tests in Open Circuit

**Figure 9:**
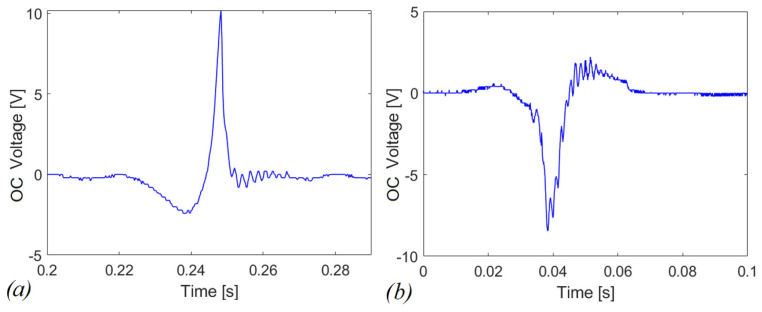
Open-circuit voltage in case of (**a**) repulsive and (**b**) attractive plucking for a single snap.

The following tests have been devised to understand the electromechanical response in the presence of magnetic plucking and indirect impact. To investigate the magnetic phenomenon, the plucking mechanism has been activated both for the repulsive and the attractive configurations with the vertical direction of motion of the moving mass (z-axis in Figure 4). However, the adopted procedure for the activation depends on the considered configuration. For the attractive case, the system is monostable, as shown in Figure 3a, and the action of the gravity field is sufficient: the moving mass is in free-fall conditions starting from the top of the slot. In the case of the repulsive scheme, the gravity force is not sufficient to overpass the potential barrier shown in Figure 3b; therefore, an additional external force is applied to move the mass along the slot. The system has been tested in two different operative conditions: (i) combined effect of magnetic plucking and indirect impacts; (ii) in the absence of impacts. The latter situation has been obtained by removing the plexiglass plate at the end of the stroke and by leaving the mass to exit the slot after the magnetic interaction phase. The obtained open circuit voltages are illustrated in Figure 9a,b for the repulsive and attractive configurations, respectively.

Despite the curves in Figure 9a,b are qualitatively similar in terms of voltage, the represented phenomena are of different nature. In the first case the system is bistable, in the second one the release is due to the overcoming of elastic restoring force offered by the piezoelectric cantilever in comparison to the nonlinear magnetic force. 

**Figure 10:**
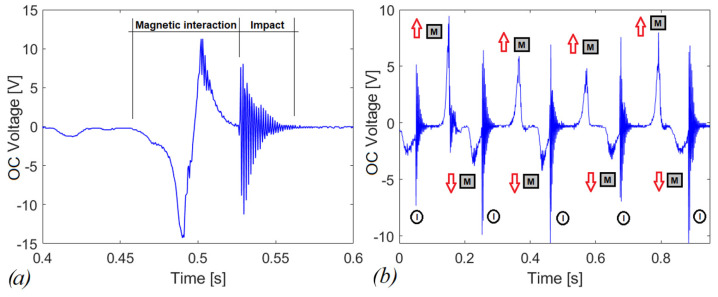
Open-circuit voltage for repulsive interaction combined with impacts (**a**) single snap (**b**) shaking with multiple snaps with indication of motion of mass (M) and impact phases.

In Figure 10a, the effect of the impact at the end of the stroke is added after the repulsive magnetic interaction. In Figure 10b, the open-circuit voltage of the piezoelectric beam is shown for shaking operation, in which a series of consecutive events, such as those shown in Figure 9a, occur. In detail more, the impacts are indicated (I in black circle) as well as the direction of motion of the moving mass *M* in time. The red upward arrow means that the mass is going up, and vice versa for the downward arrow.

### 4.3. Experimental Tests with a Connected Circuit

**Figure 11:**
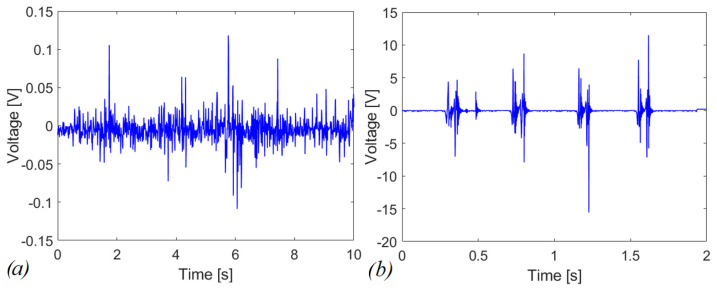
Voltage response in case of (**a**) linear system (**b**) repulsive and indirect impacts mechanisms with a load resistance of R = 100 kΩ.

The combined effect of indirect impacts and magnetic interaction is investigated in the case of connection with a load resistance R = 100 kΩ. The device has been tested for manual shaking acceleration input signal with and without the presence of the moving mass in the slot. Figure 11a shows the voltage response of the bare system (i.e., no magnetic plucking, no impact) with evidence of peak voltage less than 0.15 V. Figure 11b reports the voltage response considering the combined actuation of the magnetic plucking and the impacts. Peaks of more than 15 V in absolute value have been correctly detected. 

**Figure 12:**
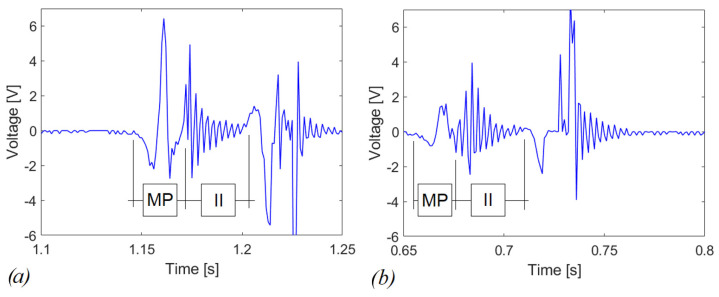
Close-up on voltage responses in case of load resistance, R = 100 kΩ (**a**) repulsive case (**b**) attractive case. MP—magnetic plucking, II—indirect impact.

This different behavior is expected since the bare system works in the linear regime and has its maximum energy-conversion effectiveness when operated at the resonance, i.e., at 665.2 Hz in the present case, but the input excitation imparted by the shaking is far from this condition. In the repulsive configuration represented in Figure 11b, 9.82 µW of average power are obtained through the Joule’s law applied to the voltage across *R*, which corresponds to an energy dissipated in the resistor of 19.63 µJ over 2 s of experiment computed by integrating the average power over time. In Figure 12a,b, a zoom of the voltage is proposed, in case of repulsive and attractive configurations, respectively. Specifically, peak voltages of 6 V and 1.5 V have been obtained in the magnetic plucking phase MP, for repulsive and attractive configurations, respectively. During the indirect-impact phase II, the magnitude of the voltage is comparable for the two configurations. Therefore, at parity of experimental conditions with comparable mechanical excitation employing the repulsive configuration, it is possible to further increase the electro-mechanical effectiveness of the piezoelectric converter compared to the attractive configuration. This is due to the bistable nature of the repulsive scheme that allows also inter-well oscillations [35].

**Figure 13:**
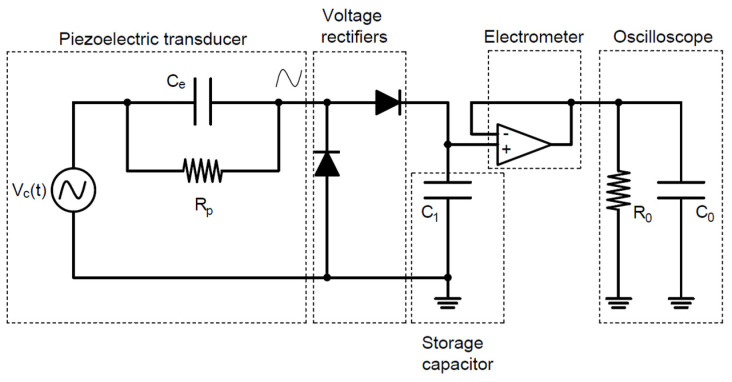
Electrical configuration of the proposed piezoelectric energy harvester.

To power the electronics within a sensor module, AC voltages provided by the piezoelectric converter must be rectified. Therefore, voltage-doubler rectifiers based on passive diodes were employed to charge a single storage capacitor C_1_ = 1 μF, as reported in Figure 13.

**Figure 14:**
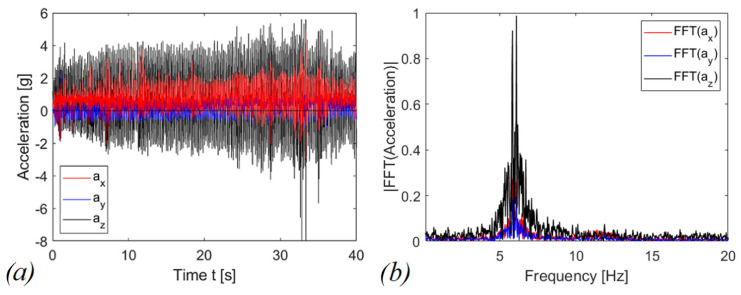
Shaking signal of (**a**) accelerogram (**b**) Fast Fourier Transform.

**Figure 15:**
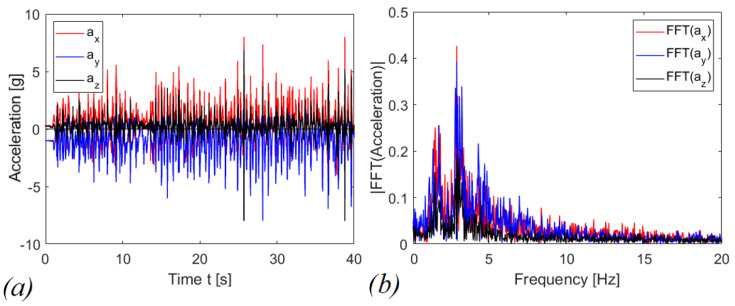
Running signal of (**a**) accelerogram (**b**) Fast Fourier Transform.

Voltage on the capacitor was acquired by employing a Keithley 6517A electrometer used as a voltage buffer with an input impedance equivalent to capacitor C_0_ = 20 pF in parallel with a resistor R_0_ > 200 TΩ. R_p_ is the internal resistance of the piezoelectric layer, generally neglected in the modelling [4]. The triaxial accelerometer and the device have been tied to the wrist of a person. Tests have been performed both in case of shaking and running activities. The shaking has been performed mainly in the direction of the guide (z axis in Figure 3) for activating the mechanism. For the running activity, instead, the motion guide has been oriented orthogonally to the axis radius of the arm as in Figure 7b. In Figure 14 and Figure 15, typical accelerograms of these events are represented with also the Fast Fourier Transforms (FFTs), for shaking and running, respectively. In the graphs, the z-component is parallel to the motion axis of the mass. The time histories of acceleration have been recorded for each different test and they are comparable in terms of the amplitude of acceleration and in frequency content. In general, the acceleration related to shaking is larger than the one for running; moreover, the dominant frequency for shaking is around 6 Hz, whereas in the case of running, the dominant frequency is around 3 Hz.

**Figure 16:**
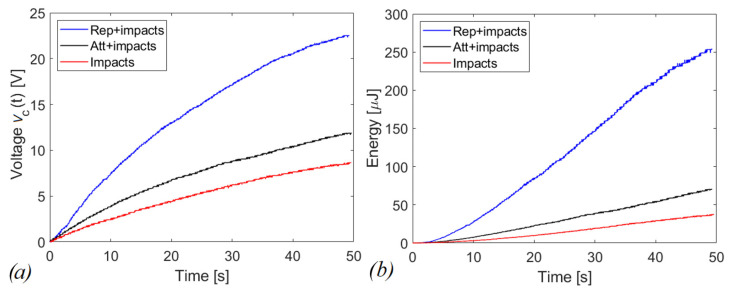
(**a**) voltage and (**b**) energy harvested in case of shaking with capacitive circuit (C_1_ = 1 µF).

**Figure 17:**
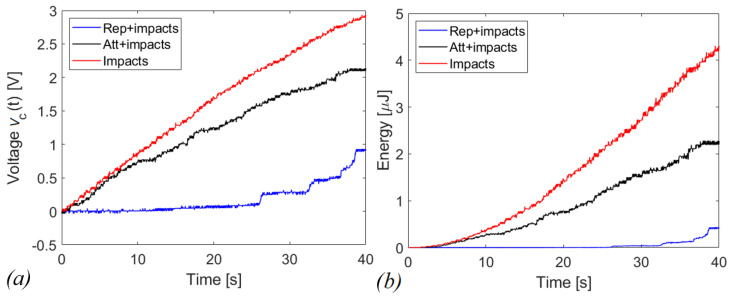
(**a**) voltage and (**b**) energy harvested in case of running with capacitive circuit (C_1_ = 1 µF).

The graphs in Figure 16 and Figure 17 report the voltages and the scavenged energy, computed through the formula:

(2) $$E=\frac{1}{2}C_{1}v_{c}{}^{2}\left(t\right)$$

in which *C*_1_ is the storage capacitance and *v_c_*(*t*) is the measured voltage. Figure 16a shows *v_c_*(*t*) as a function of time in case of shaking while Figure 16b shows the corresponding energy harvested computed through Equation (2). In this case, the magnetic interaction improves the scavenged energy (blue and black curves) in comparison to the harvester with the presence of indirect impacts only (red curve). The repulsive configuration is more promising in terms of scavenged electrical energy, at parity of experimental conditions, compared to the attractive and indirect impacts configurations, since 253.41 μJ, 70.32 μJ and 37.35 μJ have been harvested, respectively. For the case of running activity, represented in Figure 17, the behavior is reversed. Within 40 s of operation, the system recovers 0.61 μJ, 2.47 μJ, and 4.30 μJ, in the cases of repulsive, attractive, and with only indirect impacts, respectively. 

The explanation of the results in Figure 16 and Figure 17 is related to the fact that for shaking, in accordance with Figure 14, the acceleration along the guide axis is higher than the corresponding value for the running activity, represented in Figure 15. For shaking, the inertia force on the moving mass is capable to overpass the potential barrier due to magnetic interaction, so that a cycle of events is periodically repeated, with magnetic plucking and impacts of the mass. The combination of repulsive interaction and indirect impacts represents the more promising configuration in terms of output voltage, as expected in view of the abrupt snap induced when the potential barrier is overpassed.

**Figure 18:**
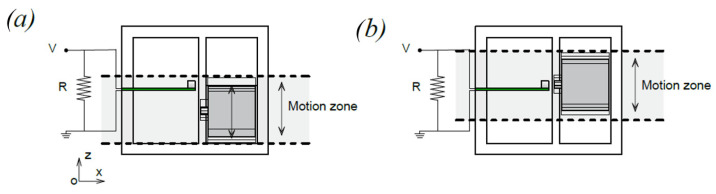
Working cycles in the case of low-level input acceleration (such as for the running activity): (**a**) repulsive interaction; (**b**) attractive interaction.

Conversely, in the case of running the input acceleration is small along the guide. The potential barrier is not exceeded, and two possible scenarios arise: in the case of repulsive configuration, due to the bistability, the mass is entrapped in the intra-well oscillation, with limited motion as shown in Figure 18a. The magnetic snap does not occur and the measured final voltage, after 40 s, is less than 2 V. In the case of attractive configuration, the moving mass is attracted by the cantilever, and then it is subjected to small non-linear oscillations around a stable configuration, as shown in Figure 18b. In this situation, the impacts are excluded. As a consequence of the continuous oscillation of the cantilever, the measured voltage is larger than the repulsive case, but smaller with respect to impacts.

The results of the experiments point out an important aspect: if the magnetic plucking occurs in addition to the indirect impacts, the benefit is guaranteed both in attractive and in repulsive configurations. In fact, despite the accelerograms being slightly different, it can be stated that in the case of shaking, the repulsive layout has a gain of about 7 times in terms of energy harvested with respect to the case of the only indirect impacts, over 50 s. In comparison with the attractive scheme, the same solution has a gain of twice as much. On the other hand, if the magnetic interaction is present but the plucking does not occur, the magnets are deleterious for the presented energy harvesting system, as experimentally demonstrated by the running activity in Figure 17.

## 5. Conclusions

This work presents a prototype of a piezoelectric nonlinear low-frequency energy harvester from vibrations and indirect impacts combined. The introduction of indirect impacts is important for the reliability of the piezoelectric components, but it is generally connected to a reduced energy transfer. It is proposed to improve the vibrational scavenging by means of magnetic plucking (i.e., physical non-linearities) through permanent magnets. Experimental investigations have been presented on the proof-of-concept prototype in different operative conditions of the mechanical input signal, magnetic interaction configurations, and electrical circuits. After experimental modal analysis, the evidence of the plucking mechanism is presented both in repulsive and in attractive configurations via external perturbation. The repulsive layout is bistable and, for this reason, the snap occurs. Differently, in the attractive layout, the snap is due to the overcome of the elastic restoring force in comparison to the magnetic force at a certain point of the dynamic interaction. The device is also tested in case of a resistive load of 100 kΩ, by comparing the voltage output for shaking signal at low frequencies (2–7 Hz) for the linear cantilever with a tip mass and the same system with the addition of indirect impacts and repulsive magnetic plucking. Finally, the harvester is used to charge a storage capacitor (1 μF) after a diode-based rectifying procedure of the output voltage. More specifically, the device has been tested without magnetic interaction (i.e., only indirect impacts effect) and with permanent magnets in repulsive and attractive configurations, in case of typical shaking and running activities. The experimental results show different behavior for different user activities. An enhancement of the performance in terms of energy is exhibited in case of combined effects of indirect impacts and magnetic interaction for shaking. This is due to the important role played by the energy of the moving magnet (i.e., the moving mass) that affects the possibility to overpass the potential barrier and to induce the magnetic plucking mechanism. If plucking does not occur, the magnetic interaction decreases the energy harvested in the system in comparison with the impact-based device. The conclusion is that the combination of magnetic plucking and indirect impacts is effective and promising, but the device should be properly engineered in order to induce the correct working cycle. This means that the magnetic force should be tuned with reference to the available external acceleration, that must be sufficient to overpass the magnetic barrier and to trigger the bistable behavior and the subsequent impacts. The design task can be tackled by means of the model presented in Equation (1), provided that the magnetic force is suitably modeled, on the basis of experimental or computational data [30]. The result of this work paves the way for developing reliable and effective energy harvesters for innovative battery-free smart sensors, in the presence of low-frequency motion.

## Figures and Tables

**Table 1:** Physical parameters and dimensions of the piezoelectric beam.

Material	ρ [kg/m^3^]	E [GPa]	ν [-]	d_31_ [pC/N]	ε_33_^s^ [-]	t [mm]	Width [mm]	Length [mm]
Titanium	4500	115	0.3	-	-	0.065	1.5	15
PZT	7500	60	0.3	212	2000	0.280 per layer	1.5	15

## Data Availability

Not applicable.
